# Evolution of resistance to thyroid cancer therapy

**DOI:** 10.18632/aging.101030

**Published:** 2016-08-27

**Authors:** Carmelo Nucera

**Affiliations:** Laboratory of Human Thyroid Cancers Preclinical and Translational Research, Division of Experimental Pathology, Cancer Research Institute (CRI), Cancer Center, Department of Pathology, Beth Israel Deaconess Medical Center, Harvard Medical School, Boston, MA 02215, USA

**Keywords:** BRAFV600E papillary thyroid cancer pre-clinical model, BRAFV600E inhibitors, resistance, MCL1 somatic copy number, clonal evolution, microenvironment

The prognosis of differentiated thyroid carcinoma (DTC), of which the majority are papillary thyroid carcinoma (PTC), shows clinical diversity among patients related to age, tumor histology and size, extra-thyroidal extension, metastasis, and thyroid follicular cell avidity for iodide. First-line preferred treatment for DTC is thyroid surgery, whenever possible, followed by radioactive iodine (RAI) in selected patients, and levothyroxine therapy in all patients (Haugen and Sherman, 2013; PMID: 23575762). As for all cancers, a percentage of patients with DTC fail to respond or respond very poorly to targeted therapies (primary resistance). In addition, many tumors that initially respond to therapy become resistant within months or even weeks (secondary resistance).

The majority of cancer patients develop resistance within ~12 months of initiation of therapy (Asik, 2016; PMID: 26364890). Despite a lot of outstanding work, much remains enigmatic about the mechanisms and development of tumor resistance. Primary resistance appears to develop early in tumorigenesis via genetic or epigenetic events that activate pro-proliferation pathways or inhibit pathways that stimulate cell death (Asik et al. 2016; PMID: 26364890). Loss or gain of a cell surface receptor or transporter or other alterations in the drug target pathway can also lead to resistance against pharmacological agents, as described below for PTC with the BRAF^V600E^ mutation.

Current evidence suggests that secondary resistance is acquired through a kind of compensatory evolution when genetic or epigenetic alterations occur or expand in the presence of targeting agents. These new genetic alterations often affect the signaling pathway targeted by the drug (Asik, 2016; PMID: 26364890). Selective drug pressure might expand specific clones that are already resistant by virtue of primarily mutated, amplified, or deleted components of the intracellular signaling pathways that inhibit tumor cell death or enhance tumor cell survival and intravascular invasion in the tumor microenvironment. Alternatively, drug treatment may actually induce alterations in DNA repair genes or other changes that result in resistance. Additional possible causes of resistance are poor drug absorption and/or abnormal drug metabolism. Properties of the tumor microenvironment can also fuel treatment resistance (Jain, 2013; PMID: 23669226). Elucidating the molecular mechanisms of both primary and secondary drug resistance is likely to identify important new therapeutic directions for combatting acquired resistance to cancer drugs.

BRAF^V600E^ is the most common mutation in PTC [1], occurring in about 60% of PTC tumors, and has been described as a clonal event since it occurs in the majority of tumor cells [1]). BRAF^V600E^ PTC exhibits primary resistance to RAI treatment, higher rates of tumor recurrence and metastases, and lower survival rates [2]. Remarkably, the BRAF^V600E^ mutation not only promotes thyroid tumor cell proliferation, adhesion, migration and invasion [3], but also up-regulates epigenetic pathways that silence expression of the sodium/iodide symporter [4]. This blocks iodide uptake, which may be one cause of primary resistance to RAI. Present in other cancers, including 40-70% of malignant melanomas and 10% of colorectal cancers, BRAF^V600E^ positive tumors provide one important case study for the evolution of drug resistance.

Recent papers reveal several different mechanisms for resistance in BRAF^V600E^ thyroid tumor cells. My group investigated one mechanism of primary resistance to orally available selective inhibitors of BRAF^V600E^ (e.g. vemurafenib) in metastatic BRAF^V600E^-PTC cells. We found that MCL1 (myeloid cell leukemia 1, chromosome 1q) copy number gain is associated with resistance to vemurafenib treatment in metastatic BRAF^V600E^-PTC cells. MCL1, an anti-apoptotic member of the BCL2 family, is amplified in many cancers and plays a crucial role in tumor progression and metastasis, and likely in drug resistance. Combining vemurafenib therapy with BCL2/MCL1 inhibitor increased death of metastatic BRAF^V600E^-PTC cells compared to single agent treatment [5]. Clinical co-treatment with BRAF^V600E^ and BCL2 inhibitors might therefore overcome intrinsic resistance to BRAF inhibitors, as observed in melanoma cells (Haq et al. 2013; PMID: 23447565).

The second paper demonstrates that the rebound in extracellular signal-regulated kinase (ERK) in the presence of vemurafenib in anaplastic or poorly differentiated human thyroid cancer cells is accompanied by increased HER3 signaling caused by induction of HER3 transcription [6]. This study provides the first rationale for combining MAP kinase pathway inhibitors (e.g. BRAF^V600E^ inhibitors, MEK inhibitors) and HER signaling inhibitors such as lapatinib, which prevents phospho-ERK1/2 rebound and sensitizes BRAF^V600E^-positive anaplastic thyroid cancer cells to BRAF^V600E^ inhibitors [6]. In the third paper, Danysh et al. showed that development of secondary resistance following long-term vemurafenib treatment in a PTC model was coincident with a spontaneous KRAS G12D mutation [7]. Activation of AKT, ERK1/2, and EGFR were observed in this model. In addition, the resistant cells were less sensitive to combinations of vemurafenib and MEK1 inhibitor or AKT inhibitor [7]. As KRAS is well known to be undruggable, it will be crucial to identify KRAS-dependent downstream targets that induce PTC cell survival in order to identify druggable targets for KRAS mutated thyroid tumors.

Finally, Gunda et al. showed that treatment of anaplastic human thyroid cancer cells using a triple drug combination - lexatumumab (TRAIL-R2 agonist antibody), the BRAF^V600E^ inhibitor PLX4720, and the PI3K inhibitor LY294002 sensitizes the cells by triggering both the extrinsic and intrinsic apoptotic pathways *in vitro* and *in vivo* [8]. Overall, these results suggest that targeting the death receptor pathway in undifferentiated thyroid cancer is a promising strategy for inducing apoptosis in thyroid cancer cells, although combination with other kinase inhibitors may be needed in aggressive tumors initially resistant to apoptosis [8].

Evidence suggests that the development of drug resistance during treatment with targeted therapy is not an aberration, but rather a predictable ‘evolution’ of tumor cells that occurs with targeted drug treatment in the vast majority of aggressive solid tumors, including thyroid cancers. Thus the prevailing approach of trying individual drugs sequentially may be actively harmful in some clinical cases. Single drug treatment of genomically heterogeneous PTC may promote clonal expansion of existing resistant tumor cells or engage other secondary resistance pathways, jeopardizing the ability of additional drugs to curb proliferation. Testing combined targeted therapy in pre-clinical models of PTC is an important step towards developing more effective therapeutic strategies for metastatic PTC refractory to standard treatments. Genomic profiling of tumors to determine an appropriate cocktail of drugs to use as second-line therapy following first-line treatment by thyroid surgery might be a valuable strategy for avoiding both primary and secondary resistance to BRAF^V600E^ inhibitors and other anti-cancer drugs.
